# Food consumption, nutrient intake, and dietary patterns in Ghanaian migrants in Europe and their compatriots in Ghana

**DOI:** 10.1080/16546628.2017.1341809

**Published:** 2017-07-06

**Authors:** Cecilia Galbete, Mary Nicolaou, Karlijn A. Meeks, Ama de-Graft Aikins, Juliet Addo, Stephen K. Amoah, Liam Smeeth, Ellis Owusu-Dabo, Kerstin Klipstein-Grobusch, Silver Bahendeka, Charles Agyemang, Frank P. Mockenhaupt, Erik J. Beune, Karien Stronks, Matthias B. Schulze, Ina Danquah

**Affiliations:** ^a^ Department of Molecular Epidemiology, German Institute of Human Nutrition Potsdam-Rehbruecke, Nuthetal, Germany; ^b^ Department of Public Health, Academic Medical Center/University of Amsterdam, Amsterdam, The Netherlands; ^c^ Regional Institute for Population Studies, University of Ghana, Legon, Ghana; ^d^ Department of Non-communicable Disease Epidemiology, Faculty of Epidemiology and Population Health, London School of Hygiene and Tropical Medicine, London, UK; ^e^ Institute of Tropical Medicine and International Health, Charité – Universitaetsmedizin, Berlin, Germany; ^f^ Faculty of Science, Kwame Nkrumah University of Science and Technology, Kumasi, Ghana; ^g^ Julius Global Health, Julius Center for Health Sciences and Primary Care, University Medical Center Utrecht, Utrecht, The Netherlands; ^h^ Division of Epidemiology and Biostatistics, School of Public Health, Faculty of Health Sciences, University of the Witwatersrand, Johannesburg, South Africa; ^i^ International Diabetes Federation, Africa Region, Kampala, Uganda

**Keywords:** RODAM, dietary patterns, sub-Sahara African populations, principal component analysis, nutrition transition, nutrient intake, diet

## Abstract

**Background**: West African immigrants in Europe are disproportionally affected by metabolic conditions compared to European host populations. Nutrition transition through urbanisation and migration may contribute to this observations, but remains to be characterised.

**Objective**: We aimed to describe the dietary behaviour and its socio-demographic factors among Ghanaian migrants in Europe and their compatriots living different Ghanaian settings.

**Methods**: The multi-centre, cross-sectional RODAM (Research on Obesity and Diabetes among African Migrants) study was conducted among Ghanaian adults in rural and urban Ghana, and Europe. Dietary patterns were identified by principal component analysis.

**Results**: Contributions of macronutrient to the daily energy intake was different across the three study sites. Three dietary patterns were identified. Adherence to the ‘mixed’ pattern was associated with female sex, higher education, and European residency. The ‘rice, pasta, meat, and fish’ pattern was associated with male sex, younger age, higher education, and urban Ghanaian environment. Adherence to the ‘roots, tubers, and plantain’ pattern was mainly related to rural Ghanaian residency.

**Conclusion**: We observed differences in food preferences across study sites: in rural Ghana, diet concentrated on starchy foods; in urban Ghana, nutrition was dominated by animal-based products; and in Europe, diet appeared to be highly diverse.

## Introduction

Ethnic minorities and migrant populations in Europe and the US are disproportionally affected by obesity and metabolic conditions, such as diabetes, hypertension and cardiovascular disease when compared with the host populations [1–4]. Moreover, these conditions are spreading globally, particularly in Africa, where the numbers are increasing rapidly [5,6]. For example, the International Diabetes Federation (IDF) estimated that in the African region the number of adults affected by diabetes will more than double within the next 35 years, from 14.2 million to 34.2 million [7]. Lifestyle modification, including smoking cessation, increasing physical activity and adopting a healthy diet is the most promising approach for diabetes prevention [8,9].

In Sub-Saharan Africa (SSA), and particularly in West African populations, ageing and rapid urbanisation are associated with lifestyle changes, including diet, contributing to the emergence of metabolic diseases [10,11]. Dietary changes in low- and middle-income countries from a more traditional to a westernised diet are universally termed as nutrition transition. Rapid economic growth leads to changes in food processing and availability, partly contributing to nutrition transition in these countries [12]. Migrants experience dietary adaptations in an even shorter time span, because migration results in a sudden change of context and thereby leading to altered dietary habits [13].

Still, data characterising the nutrition transition in West African populations are scarce, particularly for migrants in Europe [14–19]. Several approaches are available to bridge this knowledge gap spanning from food-based methods, to nutrient analysis, up to the identification of dietary patterns.

The latter appears ideal to capture the complexity of human dietary behaviour, preferably using evidence-based scores or exploratory techniques [20]. The *a priori* approach is based on nutritional recommendations and established diet-disease relationships to calculate pre-defined dietary patterns scores from the intake of certain food groups and nutrients. In comparison, the *a posteriori* approach constitutes an exploratory method which is purely data-driven and hypothesis-free [21].

Contrasting the upsurge of obesity and metabolic conditions in West African populations, and the potential importance of nutritional changes for this development, dietary habits of West Africans in Europe and in their home countries remain to be characterised. Thus, we aimed at investigating dietary behaviour among a homogeneous group of West Africans who live in or originate from the Ashanti Region of Ghana. The specific objectives were to examine food consumption, to analyse the intakes of energy and nutrients, to identify exploratory dietary patterns, and to investigate socio-demographic factors of pattern adherence among Ghanaians living in rural Ghana, urban Ghana, and Europe (Amsterdam, London, and Berlin).

## Material and methods

### Study design and population

**Figure 1.**
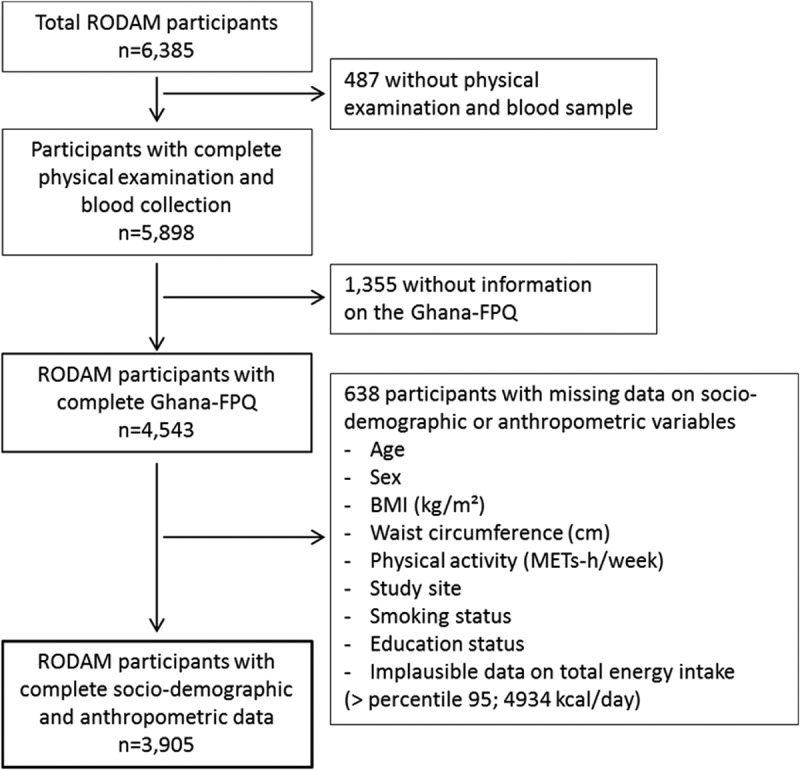
Flow-chart of excluded RODAM study participants because of missing or implausible data. The exclusion of those participants with total energy intake > percentile 95 (4934 kcal/day) allowed to control for normality. The 1355 participants without information on the Ghana-FPQ include participants in which this was not conducted (n = 1,262), and participants with the whole questionnaire or one or more whole sections blank (*n* = 93). FPQ: Food Propensity Questionnaire.

The detailed objectives and procedures of the multi-centre, cross-sectional RODAM (Research on Obesity and Diabetes among African Migrants) study have been published elsewhere [22]. In brief, we recruited 6385 Ghanaian adults, aged ≥18 years living in urban Ghana (Kumasi and Obuasi, *n* = 1619), rural Ghana (Ashanti Region, *n* = 946), Amsterdam (*n* = 1900), London (*n* = 1258), and Berlin (*n* = 662). The primary aim of the RODAM study was to identify the relative contributions of demographic, socio-economic, psycho-social, lifestyle and (epi)genetic risk factors for obesity and type 2 diabetes in this West African population. Data collection comprised an extensive general questionnaire applied by trained personnel, a series of qualitative interviews regarding knowledge, attitudes, and practices, and a detailed documentation of the dietary habits. Further, physical examinations were conducted and biological samples (fasting blood, urine) were drawn for biochemical, genetic, and epigenetic analysis. Ethical approval was obtained from the local ethics committees at all study sites and all participants gave informed written consent. Figure 1 presents the flow diagram of excluded participants because of missing or implausible data, resulting in a sample size of 4543 participants for the characterisation of dietary behaviour and a sample size of 3905 participants for the examination of socio-demographic factors of pattern adherence.

### Nutritional assessment

The methods of dietary assessment and calculations of energy and nutrient intakes are provided in Supplementary Figure 1. At all RODAM study sites, food intake was assessed with a standardised Food Propensity Questionnaire (Ghana-FPQ) that queries for the usual intake frequencies of food groups in the preceding 12 months. The Ghana-FPQ covers 134 items and is based on the multi-language, semi-quantitative European Food Propensity Questionnaire (EFPQ) [23]. In addition, we incorporated typical Ghanaian foods that were identified in the Ghana Demographic and Heath Survey (2008, [24]) and in previous studies among Ghanaians in Amsterdam (the GHAIA study [25]) and urban Ghana [15]. Common Ghanaian household utensils facilitated standardised description of portion sizes [26]. To estimate the usual daily intake of foods in grams per day we combined intake frequencies with standard portion sizes. For European foods, portion sizes of the EFPQ were applied and for Ghanaian items, we conducted 24 h-dietary recalls (24hDR) in a random sub-sample (*n* = 251) to obtain portion sizes. Lastly, the German Nutrient Database (BLS 3.01) (2010) and the West African Food Composition Table (2012) were used to translate usual food intake (g/d) into energy consumption and intake of nutrients (Supplementary Figure 1).

### Assessment of socio-demographic, anthropometric, and lifestyle factors

The general questionnaire was either self-administered or applied in face-to-face interviews by trained study personnel. We obtained data on demographics, socio-economics, migration-related factors, psychosocial vulnerability, health status, and health behaviour. The assessment of educational status was adapted to local circumstances at the different study locations and comprised four categories: never been to school or elementary school; lower vocational schooling or lower secondary schooling; intermediate vocational schooling or intermediate/higher secondary schooling; and higher vocational schooling or university. Height was measured with a portable stadiometer, weight with a digital scale, and waist circumference with a measuring tape (all devices SECA, Germany). Body mass index (BMI) was calculated as weight/height^2^ (kg/m^2^). Physical activity was assessed using the WHO Global Physical Activity Questionnaire [27] and was categorised as high, moderate, or low, according to Haskell et al. [28]. Smoking status was assessed through the question ‘Do you smoke at all?’ and participants were categorised as current, former, or non-smokers.

### Identification of dietary patterns

The 134 Ghana-FPQ food items were collapsed into 30 food groups according to their culinary use and nutrient profile (Table 1). In 4543 RODAM study participants, dietary patterns were identified by means of principal component analysis (PCA), using the PROC FACTOR procedure in SAS 9.4. This procedure identifies latent factors that explain the maximum of the total variance of food intake. The factors were orthogonally rotated (varimax rotation) to facilitate the interpretability of the factors and to ensure that the factors remain uncorrelated. The decision on the number of factors that should be retained was based on the inspection of the scree plot, an eigenvalue >1, and the plausibility of the factors. Rotated factors with |factor loading| ≥ 0.30 were considered to contribute to the pattern. Every participant received a score for each of the identified factors to rank the participants according pattern adherence. This score was computed by summing up intakes of each food group weighted by its factor loading, which represents the relative contribution of that food group.

**Table 1.** Food groups used in the dietary patterns.

Food group	Food items included
Whole grain cereals	Whole grain bread, wholegrain crispbread, muesli cereals, and other grains (millet, couscous, polenta, spelt, and barley)
Refined cereals	White wheat bread, white crispbread, hot cereals, and porridge
Sweet spreads	Marmalade, jam, jelly, and honey
Dairy products	Cocoa milk drink, fruit milk drink, plain yoghurt, buttermilk, flavoured yoghurt, soft cheese, semi-soft/firm cheese, sour milk, quark, mozzarella, mascarpone, feta cheese, butter, whipped cream
Fruits	Orange, mandarin, kiwi, watermelon, mango, cantaloupe, pawpaw, pineapple, banana, plum, peach, apricot, nectarine, flat peach, apple, pear, strawberries, cherries, berries, grapes, and stewed fruit
Nuts and seeds	Dried fruit, nuts, and seeds
Roots, tubers & plantain	Plantain, cassava, yam, and fufu
Potatoes	Potatoes, pan fried potatoes, French fries, and sweet potatoes
Fermented maize products	Banku and kenkey
Vegetables	Green leaves, spinach, chard, lettuce, endive, chicory, Chinese and white cabbage, tomatoes, peppers, carrots, cucumber, eggplant, beans (green beans), onions and garlic
Legumes	Groundnut soup, legumes, lentil-pea and bean soup
Vegetable soups, stews, sauces	Palmnut soup, nkontomire stew, okro stew, tomato sauce and stew, vegetable soup
Rice and pasta	Rice, pasta, noodles, and macaroni
Egg	Egg
Red meat	Beef, goat, pork, bush meat, liver, and giblets
Poultry	Poultry
Processed meat	Meatballs, fried sausage, boiled sausage, dry and cured meat, salami, jagdwurst, bologna, mortadella, ham corned beef, liverwurst, and liver pâté
Fish	Fatty fish, lean fish, fish preparations and shellfish
Meaty mixed dishes	Lasagne, pizza and mixed dishes with meat (fufuo ne nkatenkwan)
Vegetarian mixed dishes	Mixed dishes without meat (red red, ampesie) and tofu
Cakes and sweets	Tart, pie, yeast cake, pastry, sponge cake, cream pie, cheesecake, cookies, chocolate, sweets, candy, and toffee
Coffee and tea	Regular coffee, decaffeinated coffee, black and green tea, and fruit and herbal tea
Alcoholic beverages	Regular beer, wine, liquors, and spirits
Sodas and juices	Non-alcoholic beer, sodas and minerals, light and soft drinks, fruit juices, fruit nectars, vegetable juices
Palm oil	Palm oil
Olive oil	Olive oil
Other oils	Other oils and peanut butter
Margarine	Regular margarine and fat-reduced margarine
Cooking fats	Cooking fats (e.g. animal fats like lard or speck)
Condiments	Ketchup, mayonnaise, crème fraiche, salad cream, sour cream, remoulade, and sauces

### Statistical analysis

For the RODAM study population, socio-demographic, anthropometric, and lifestyle characteristics, including daily energy consumption and nutrient intakes, are presented as mean (± standard deviation, SD) for normally distributed continuous variables and as median (IQR: interquartile range) for non-normally distributed continuous variables. Categorical variables are presented as percentages.

In 3905 participants with complete data (Figure 1), we examined the distributions of socio-demographic and anthropometric characteristics across quintiles of the pattern scores using trend test (continuous variables) and χ^2^-test (categorical variables). For non-normally distributed variables the median per quintile was subjected to the trend test. Lastly, multiple linear regression models with a backward elimination procedure (*p* < 0.05) were calculated to identify independent socio-demographic factors of adherence to the identified dietary patterns.

## Results

### Study population

Socio-demographic, anthropometric, and lifestyle characteristics of the RODAM study population are presented in Table 2. The majority were female (63%) and middle-aged (mean, 46.5 years; SD, 11.8 years). Men were older, had a higher educational status, were more likely to be former or current smokers, were more physically active, and had lower BMI and waist circumference than women. RODAM participants in Europe had the highest degree of education, were more frequently former or current smokers and presented with higher BMI and waist circumference than their counterparts in Ghana. The mean length of stay in Europe was 16.9 (SD, 9.9 years) years. RODAM participants in rural Ghana had the lowest degree of education, were physically more active than those in urban Ghana and Europe, and had the lowest BMI and waist circumference.

**Table 2.** Socio-demographic and anthropometric characteristics of the RODAM study participants.

	All (*n* = 3905)	Men (*n* = 1449)	Women (*n* = 2456)	Rural Ghana (*n* = 926)	Urban Ghana (*n* = 1367)	Europe (*n* = 1612)
Sex (% male)	37.1	-	-	39.1	27.9	43.7
Age (years)	46.5 (11.8)	47.5 (12.2)	45.9 (11.6)	48.6 (14.3)	45.4 (11.5)	46.4 (10.4)
Years in Europe^+^	16.9 (9.9)	17.1 (10.3)	16.8 (9.5)	-	-	16.9 (9.9)
Study site (%)						
Europe	41.3	48.7	36.9	-	-	100.0
Urban Ghana	35.0	26.4	40.1	-	100.0	-
Rural Ghana	23.7	25.0	23.0	100.0	-	-
Education (%)						
Never or elementary	38.3	23.0	47.4	59.1	44.0	21.6
Low	36.9	40.8	34.7	29.9	38.8	39.3
Intermediate	16.1	22.1	12.6	7.6	12.4	24.1
Higher vocational (university or schooling)	8.7	14.2	5.4	3.5	4.8	15.0
Smoking (% current or former)	9.5	19.9	3.3	8.6	6.8	12.2
Total Energy intake (kcal/day)	2528 (840)	2619 (858)	2475 (824)	2611 (848)	2295 (660)	2677 (924)
Carbohydrates intake (energy %)	53.3 (9.1)	52.6 (9.4)	53.7 (8.9)	56.5 (8.3)	54.4 (8.1)	50.4 (9.5)
Fat intake (energy %)	32.3 (8.3)	32.2 (8.6)	32.4 (8.1)	31.3 (7.3)	31.6 (7.3)	33.5 (9.4)
Protein intake (energy %)	13.4 (2.7)	13.4 (2.6)	13.4 (2.7)	11.5 (2.2)	13.6 (2.5)	14.4 (2.5)
Alcohol (g/day)*	0.12 (0,1.83)	0.77 (0,5.1)	0.06 (0,1.02)	0.06 (0,1.22)	0.06 (0,0.64)	0.85 (0,4.7)
Physical activity (METs-h/week)*	70 (14,168)	96 (26,196)	57 (10,155)	88 (32,161)	60 (6,156)	62 (14,186)
BMI (kg/m^2^)	26.6 (5.5)	24.7 (4.5)	27.7 (5.8)	22.5 (4.3)	26.9 (5.4)	28.6 (5.0)
Waist circumference (cm)	89.4 (12.6)	86.7 (12.2)	91.0 (12.6)	81.2 (10.9)	89.4 (11.8)	94.1 (11.7)

Data are shown as mean (standard deviation. * Data are shown as median (percentile 25, percentile 75).

^+^ Sample size for the variable ‘Years in Europe’: *n* total = 1536; men, *n* = 667; women, *n* = 862.

### Intakes of energy, nutrients, and food groups

The daily intakes of energy (kcal/day) and macronutrients (energy %) are presented in Table 2. Mean total estimated energy intake was higher in men than in women. On average, estimated energy intake of Ghanaians residing in Europe (mean ± SD kcal/day: 2677 ± 660) was higher as estimated energy intake in rural (2611 ± 848) and urban Ghana (2295 ± 660). In the total population carbohydrates, total fat, and protein contributed 53%, 32%, and 14% to the daily energy intake, respectively. This was similar between men and women, but was distinct across study sites: In Europe, energy percentages were shifted towards protein and total fat; in urban Ghana, carbohydrates supplied most of the daily energy; and in rural Ghana, energy intake from carbohydrates was even more pronounced.

**Figure 2.**
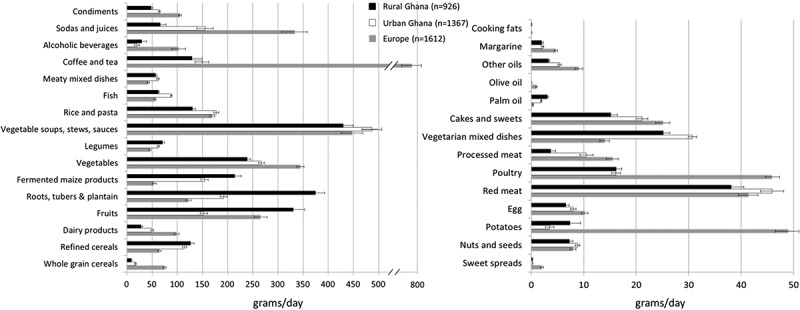
Mean intakes and standard deviation (g/day) of 30 food groups according to RODAM study site (*n* = 3905). (a) Food groups with a mean intake of >50 g/day. (b) Food groups with a mean intake of ≤50 g/day.

In Figure 2, we present the mean intakes of food groups (g/day) according to the RODAM study sites rural Ghana, urban Ghana and Europe, separately for food groups. In Europe, the daily intakes of condiments, sodas and juices, coffee and tea, vegetables, dairy products, sweet spreads, whole grain cereals, and alcoholic beverages were higher than in urban Ghana, followed by rural Ghana (Figure 2(a)). The opposite trend was observed for refined cereals, fermented maize products, and roots, tubers, and plantain. The intakes of vegetable soups and stews, rice and pasta, and meaty mixed dishes were similar across study sites (Figure 2(a)). For food groups with a mean intake of ≤50 g/day (Figure 2(b)), the consumption of palm oil was nine times lower in Europe than in rural Ghana. Olive oil was consumed only in Europe, while margarine was also consumed in Ghana. The intake of potatoes was highest in Europe followed by rural Ghana, and urban Ghana. Cakes and sweets and processed meat were most frequently consumed in Europe than in urban Ghana, followed by rural Ghana. Consumption of red meat was similar across the three study sites.

### Exploratory dietary patterns

**Figure 3.**
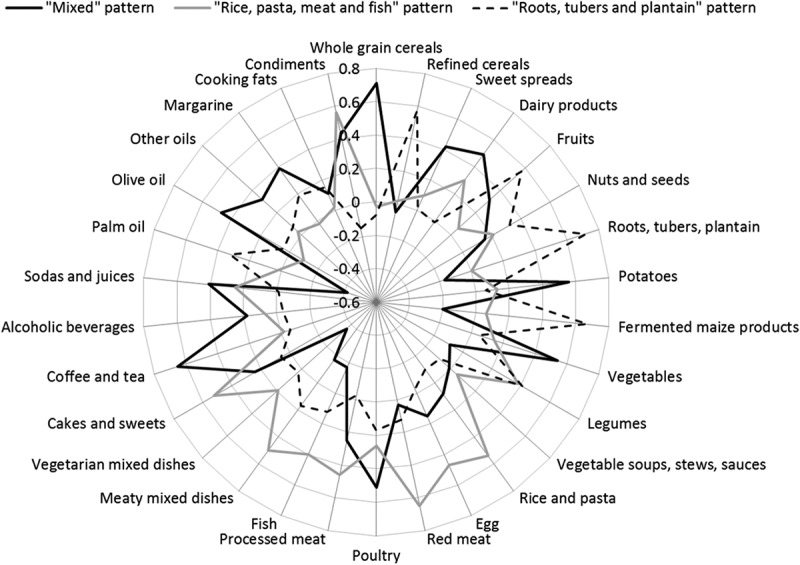
Dietary patterns derived by principal component analysis and rotated factor loadings in 4543 Ghanaians. Solid black line, the ‘mixed’ pattern, was characterised by high intakes of whole grain cereals, sweet spreads, dairy products, potatoes, vegetables, poultry, coffee and tea, sodas and juices, olive oil, other oils and margarines. Grey line, the ‘rice, pasta, meat, and fish’ pattern, was characterised by high intakes of legumes, rice and pasta, egg, red meat, processed meat, fish, meaty mixed dishes, cakes and sweets, sodas and juices, and condiments. Dashed black line, the ‘roots, tubers, and plantain’ pattern, was characterised by high intakes of refined cereals, fruits, nuts and seeds, roots, tubers and plantain, fermented maize products, legumes, and palm oil.

By means of PCA we identified three dietary patterns explaining 29% of the total variance in food intake. Because the identified dietary patterns were almost identical for both sexes, we applied PCA to the total study population (data not shown). Figure 3 displays the identified dietary patterns and their rotated factor loadings as a spider web chart. The first factor, named ‘mixed’ pattern, was characterised by high intakes of whole grain cereals, sweet spreads, dairy products, potatoes, vegetables, poultry, coffee and tea, sodas and juices, olive oil, margarine, and condiments, and by low intakes of vegetarian mixed dishes and palm oil (Figure 3). This ‘mixed’ pattern explained 14.4% of the total variance in food intake. Participants in Europe exhibited the highest median score for this pattern (0.73, IQR: −0.34 to 1.21) (Supplementary Table 1). The second extracted factor, labelled ‘rice, pasta, meat, and fish’ pattern, accounted for 8.8% of the total variance in food intake and was characterised by high intakes of dairy products, red meat, processed meat, eggs, legumes, rice and pasta, fish, meaty mixed dishes, and cakes and sweets, and condiments (Figure 3). Participants in urban Ghana had the highest median score for this ‘rice, pasta, meat and fish’ pattern (0.13, IQR: −0.40 to 0.79) (Supplementary Table 1). Finally, we identified a third factor, called ‘roots, tubers, and plantain’ pattern that accounted for 5.7% of the total variance in food intake. This patterns was characterised by high intakes of refined cereals, fruits, nuts and seeds, roots, tubers and plantain, fermented maize products (banku and kenkey), legumes, and palm oil (Figure 3). Participants in rural Ghana showed the highest median score for this pattern (0.49, IQR: −0.01 to 1.28) (Supplemenary Table 1).

After exclusion of participants who reported dietary changes within the last 12 months, we revealed similar dietary patterns by PCA. In Table 3 we present the socio-demographic, anthropometric, and lifestyle characteristics of the RODAM participants across quintiles of the three dietary patterns identified. For the ‘mixed’ dietary pattern participants in higher quintiles compared with lower quintiles had a similar age, but were more likely to be male, had a higher education, were more often former or current smokers, were more physically active, had higher BMI and waist circumference, higher total energy intake, and higher intake of dietary protein. Most of the participants adhering to this pattern lived in Europe. For the ‘rice, pasta, meat, and fish’ dietary pattern, participants in the highest quintile compared to lower quintiles did not differ in sex, smoking status and waist circumference. However, those in the highest quintile were younger and stayed shorter in Europe, exhibited more often low or intermediate educational level, were physically more active, had higher BMI and higher mean energy intake than their counterparts in lower quintiles. Half of the participants in the highest quintile of this dietary pattern lived in urban Ghana. With regard to the ‘roots, tubers, and plantain’ dietary pattern participants in the highest quintile compared to lower quintiles were more likely women and of older age; length of stay in Europe was longer and formal education was absent; they had a similar smoking behaviour, exhibited lower BMI and waist circumference, and had a higher daily energy intake. In the highest quintile of the ‘roots, tubers, and plantain’ pattern score, 57% lived in rural Ghana and 26% in urban Ghana.

**Table 3.** Socio-demographic and anthropometric characteristics across quintiles of the three dietary pattern scores identified. Only Q1, Q3, and Q5 are shown.

	‘Mixed’ pattern			‘Rice, pasta, meat, and fish’ pattern			‘’Roots, tubers, and plantain’ pattern		
	Q1 (*n* = 781)	Q3 (*n* = 781)	Q5 (*n* = 781)	Q1 (*n* = 781)	Q3 (*n* = 781)	Q5 (*n* = 781)	Q1 (*n* = 781)	Q3 (*n* = 781)	Q5 (*n* = 781)
Sex (% male)	32.3	34.8	43.2	38.0 ^c^	36.9 ^c^	38.5 ^c^	42.8	34.8	36.6
Age (years)	47.6 (12.9) ^c^	45.4 (12.1) ^c^	47.2 (10.4) ^c^	51.7 (11.8)	46.6 (11.0)	41.4 (11.1)	45.0 (11.0)	47.0 (11.7)	47.5 (12.8)
Years in Europe ^a^	16.2 (8.3)	12.1 (13.4)	18.5 (14.8)	19.4 (13.8)	15.3 (13.9)	8.7 (14.3)	14.8 (14.5)	16.5 (14)	19.8 (17.0)
Study site (%)									
Europe	0.3	18.3	99.0	45.7	42.8	37.2	78.6	34.7	17.8
Urban Ghana	57.0	48.1	0.3	18.6	35.5	49.2	19.5	46.0	25.9
Rural Ghana	42.8	33.6	0.8	35.8	21.8	13.7	1.9	19.3	56.3
Education (%)									
Never or elementary	49.9	43.0	23.1	45.8	38.9	26.9	25.7	38.8	48.5
Low	38.5	36.8	35.9	30.7	36.0	42.8	39.1	37.8	32.9
Intermediate	7.9	13.6	24.8	15.3	16.7	20.4	22.8	16.3	11.3
Higher vocational	3.6	6.7	16.3	8.2	8.5	10.0	12.4	7.2	7.3
Smoking (% current or former)	7.3	7.7	14.1	10.5 ^c^	9.1 ^c^	11.3 ^c^	11.0 ^c^	9.4 ^c^	10.0 ^c^
Total Energy intake (kcal/day)	2195 (640)	2613 (819)	2941 (898)	2187 (856)	2392 (727)	3092 (758)	2377 (864)	2344 (740)	3144 (786)
Carbohydrates intake (energy %)	52.4 (8.0)	56.0 (9.1)	52.2 (8.9)	55.3 (10.7)	53.4 (8.5)	50.9 (7.9)	48.4 (9.6)	53.4 (8.1)	58.6 (8.2)
Fat intake (energy %)	33.9 (7.3) ^c^	30.6 (8.1) ^c^	31.8 (8.2) ^c^	31.8 (9.6) ^c^	32.0 (7.9) ^c^	33.0 (7.0) ^c^	35.3 (10.0)	32.4 (7.6)	28.7 (6.7)
Protein intake (energy %)	13.2 (2.8)	12.7 (2.6)	14.2 (2.4)	12.0 (2.9)	13.5 (2.4)	15.0 (2.3)	14.7 (2.7)	13.4 (2.5)	12.1 (2.4)
Alcohol intake (g/day) ^b^	0.06 (0, 0.77)	0.06 (0, 1.18)	1.30 (0, 6.33)	0.06 (0, 1.47)	0.12 (0, 1.91)	0.58 (0, 2.89)	0.58 (0, 3.26)	0.12 (0, 1.51)	0.06 (0, 1.44)
Physical activity (METs-h/week) ^b^	99 (24, 183)	52 (10, 136)	72 (17, 212)	56 (10, 148)	74 (12, 174)	90 (24, 184)	80 (11, 203)	69 (13, 162)	76 (22, 156)
BMI (kg/m^2^)	24.9 (5.5)	26.0 (5.7)	28.5 (5.1)	25.7 (5.5)	26.8 (5.6)	26.9 (5.3)	28.3 (5.2)	26.8 (5.5)	24.4 (5.2)
Waist circumference (cm)	85.7 (12.5)	87.9 (12.5)	93.9 (12.0)	88.5 (12.3) ^c^	89.6 (13.0) ^c^	89.4 (12.1) ^c^	93.0 (12.6)	90.0 (12.7)	85.1 (11.9)

Data are shown as mean (standard deviation) unless otherwise stated. *p-*values for trend were calculated within each dietary pattern for quantitative variables and overall *p*-values were calculated by χ^2^-test for categorical variables.

^a^Sample size for the variable ‘Years in Europe’: Q1, *n* = 2; Q3, *n* = 136; Q5, *n* = 734 for the ‘mixed’ pattern; Q1, *n* = 336; Q3, *n* = 324; Q5, *n* = 280 for the ‘rice, pasta, meat, and fish’ pattern and; Q1, *n* = 588; Q3, *n* = 260; Q5, *n* = 126 for the ‘roots, tubers, and plantain’ pattern.

^b^Data are shown as median (percentile25, percentile75).

^c^Reflects not significant *p*-values (*p* ≥ 0.05).

Supplementary Tables 2, 3, and 4 show the fibre and micro-nutrient intakes (per 1000 kcal) through quintiles of adherence to the ‘mixed’, ‘rice, pasta, meat, and fish’, and ‘roots, tubers, and plantain’ patterns, respectively. Higher adherence to the ‘mixed’ pattern was associated with higher intakes of Ca, Fe, Mg, P, K, thiamine, riboflavin, niacin, and vitamin E, and lower intakes of vitamins B_12_ and D. Similarly, higher adherence to the ‘rice, pasta, meat, and fish’ pattern was related with higher intakes of Na and P, niacin, and vitamins B_12_ and D, and lower intakes of fibre, Mg, K, retinol equivalents, folate, and vitamin C. For the ‘roots, tubers, and plantain’ pattern, a high adherence was associated with higher intakes of fibre and vitamin C, and lower intakes of Ca, Fe, P, Na, K, Zn, Cu, retinol equivalents, riboflavin, niacin, and vitamin E.

### Factors of adherence to dietary patterns

Lastly, we identified independent socio-demographic factors of adherence for the identified dietary patterns. Table 4 shows the results of multiple linear regression models using a backward elimination procedure. Adherence to the ‘mixed’ pattern was associated with European residence (reference: rural Ghana), high education (reference: low education), current or former smoking (reference: never), and female sex (reference: male). These factors explained 69% of the variance of the ‘mixed’ dietary pattern score. With respect to the ‘rice, pasta, meat, and fish’ pattern, adherence was associated with residence in urban Ghana, European residence, current or former smoking, high education, male sex, high physical activity, and younger age. These variables explained 15% of the variance of the ‘rice, pasta, meat, and fish’ pattern score. Regarding the ‘roots, tubers, and plantain’ pattern, independent factors of adherence were residence in rural Ghana and older age. These variables accounted for 27% of the total variance of the ‘roots, tubers, and plantain’ pattern score.

**Table 4.** Independent factors of adherence to dietary patterns (*n* = 3905).

	β	95% CI	*p* value
MIXED PATTERN (r^2^ = 68.8%)			
Sex (women vs. men)	0.052	0.012, 0.093	0.012
Smoking (former/current vs. never)	0.122	0.056, 0.187	< 0.001
Education (high vs. low)	0.120	0.075, 0.166	< 0.001
Study sites (Europe vs. rural Ghana)	1.609	1.556, 1.654	< 0.001
Study site (Urban Ghana vs rural Ghana)	−0.007	−0.056, 0.043	0.797
RICE, PASTA, MEAT, AND FISH PATTERN (r^2^ = 14.5%)			
Sex (women vs. men)	−0.066	−0.130, −0.001	0.048
Age (years)	−0.024	−0.027, −0.022	< 0.001
Smoking (former/current vs. never)	0.198	0.095, 0.302	< 0.001
Education (high vs. low)	0.142	0.070, 0.214	<0.001
Physical activity (categorised*)	0.047	0.012, 0.081	0.008
Study sites (Europe vs. rural Ghana)	0.148	0.071, 0.226	<0.001
Study site (Urban Ghana vs rural Ghana)	0.530	0.451, 0.608	< 0.001
ROOTS, TUBERS, AND PLANTAIN PATTERN (r^2^ = 27.2%)			
Age	0.002	0.000, 0.005	0.046
Study sites (Europe vs. rural Ghana)	−1.334	−1.403, −1.265	< 0.001
Study site (Urban Ghana vs rural Ghana)	−0.867	−0.939, −0.796	< 0.001

Beta coefficients (β), 95% confidence intervals (CIs) and *p*-values were calculated by multiple linear regression models, including all factors listed for each pattern. Independent factors of adherence to each pattern were identified using backward elimination procedure.

* Physical activity was categorised into three levels: low, moderate, and high, according to Haskell et al. [28].

## Discussion

In this cross-sectional study among a large sample of middle-aged Ghanaian men and women in Europe and their compatriots in Ghana, we assessed dietary behaviour by means of culture-specific instruments and observed differences across the study sites. The diet in Ghana – specifically in rural areas – relied more on simple carbohydrates and traditional foods, whereas the diet in Europe appeared to be less starch-based and more diversified. Mainly, three dietary patterns, differentially associated with socio-demographic factors, were identified. While adherence to the ‘mixed’ patterns was more associated with female sex and European residence, the ‘rice, pasta, meat, and fish’ pattern was associated with male sex, younger age, more physical activity, and residence in urban Ghana but also in Europe. Both patterns were associated with higher education and current or former smoking status. The described ‘roots, tubers, and plantain’ pattern was mainly characterised by residence in rural Ghana and slightly associated with higher age.

### RODAM dietary behaviour and nutrition transition

In 1993, Barry Popkin coined the term ‘nutrition transition’ [29]. This theory assumes that human diet changes over time and alongside economic development [29]. Within the RODAM population we have observed distinctions between Ghanaians living in Europe, urban Ghana, and rural Ghana with respect to their macro-nutrient consumptions, arguing for the presence of a nutrition transition in West African populations who are facing rapid environmental changes. We observed that in Ghana the diet was richer in carbohydrates than that in Europe, and this was also true for rural Ghana in comparison with urban Ghana. The inverse tendency was discernible for fat and protein intake. Indeed, similar nutrient distributions between rural and urban areas were reported from Cameroon where the intakes of protein and saturated fats were higher in urban places than in rural areas. Yet, this trend was less clear-cut for carbohydrates and total fat [30]. Also, different trends in the intake of fibre and micro-nutrients were observed for the three different dietary patterns identified.

These differences in macro- and micronutrient intakes may reflect the differences in food group consumption, which additionally corroborate the phenomenon of nutrition transition upon urbanisation and migration. We observed a trend towards higher intakes of westernised foods, such as condiments, sodas and juices, and cakes and sweets in Europe followed by urban Ghana and rural Ghana. Still, the same was seen for food groups with proved beneficial health effects, including whole grain cereals and vegetables. The opposite trend was discernible for typical Ghanaian fermented maize products, palm oil, and roots, tubers, and plantain.

So far, in West African populations, only few studies in mainly urban areas have been conducted to identify dietary patterns by exploratory analysis [14,15,17–19]. Most of them described the presence of a labelled ‘traditional’ pattern [15,18,19]. Sodjinou et al. described this ‘traditional’ pattern as high in grains and cereals [18] and Zeba et al. as high in local cereals, legumes, and traditional green leafy vegetables [19]. The ‘traditional’ pattern identified by Frank et al. was characterised by high intakes of plantain, green leafy vegetables, beans, garden egg, fish, maize (banku), palm oil, okra and fruits, quite similar to our described ‘roots, tubers, and plantain’ pattern [15]. Moreover, this ‘roots, tubers, and plantain’ pattern accords with the previously described traditional Ghanaian diet consisting of a main energy dense component (yams, maize, millet, black-eyed peas, maize, cassava, yams, cocoyam, and plantains) served with either a soup or a stew [31]. Previous studies also identified a more diversified dietary pattern that could be interpreted as a synthesis of the identified ‘mixed’ pattern and ‘rice, pasta, meat, and fish’ pattern. Another study conducted in Yaoundé, Cameroon, described two patterns [17]: the ‘fruit and vegetables’ pattern, typified by high intakes of fruits, vegetables, tubers, and legumes, and the ‘meat’ pattern, characterised by high intakes of bush meat, poultry, and red meat. Similarly, in Ouagadougou, Burkina Faso, a meaty pattern named as ‘modern food’ pattern was identified that was rich in meats and poultry, eggs, and processed meat, The second pattern was named ‘snacking’ and was characterised by high intakes of fried foods, vegetable source fats, sugar and sweetened products and drinks, cereals, vegetables, dairy products, non-fatty meats and poultry, fresh fish, and roots and tubers. Clearly, comparing food patterns across Sub-Saharan African populations is challenging due to the different nature of the applied exploratory methods. Furthermore, the extraction of the patterns involves subjective decisions from the formation of the food groups to the number of factors that are finally retained.

The dietary behaviour of the RODAM participants based in Europe suggests that the new European scene offers a wide variety of new products and choices, leading to changes in their usual diet. Something similar was observed in a study among 213 migrants from Equatorial Guinea (Bubis) living in Madrid; the diet was richer in protein intake and lower in fat than the original Guinean diet [16]. Among the Bubis in Madrid, two dietary patterns were identified. Most participants adhered to the ‘healthier’ pattern, which was associated with a higher consumption of fish, fruits, vegetables, legumes, dairy products and bread [16]. Similar to the ‘mixed’ patterns in the present study, the ‘healthier’ pattern among Bubis was strongly related to female sex, longer duration of residence in Spain, and former smoking.

### Patterns adherence and nutrition transition

Previous studies on exploratory pattern analysis also characterised the patterns in terms of socio-demographic traits. The ‘traditional’ pattern consistently associated rural residence, with a lower income, poor education, older age, and female gender [15,18,19]. In Cameroon, Benin, and Burkina Faso, diversified patterns were related to younger age, higher socio-economic status, and higher degree of education [14,18,19].

Our results support the concept of nutrition transition through changes in the environment, due to migration or rapid urbanisation. We compared individuals of a homogeneous population from one geographical area who now live in different urbanised environments and observed differences in dietary choices and preferences across the study locations. In line with this, Micklesfield et al. proposed that areas with slower and more recent urbanisation, comparing with Europe and the US, and similar to urban Ghana, people with a higher socio-economic status and higher education are more likely to engage in a more ‘westernised’ diet [32]. However, it has been observed that in Europe higher socio-economic groups are more likely to have a higher compliance with dietary recommendations and guidelines [33–36]. Recently, Pessoa et al. proposed that the food environment, such as healthy food availability explains the socio-economic disparities with respect to food choices [37].

Some strengths and limitations of our study deserve to be mentioned. For nutritional assessment we applied a culture-specific, semi-quantitative food propensity questionnaire that has demonstrated its feasibility and good acceptance within the RODAM study population, however this has not been validated yet. While this technique is practical, affordable and widely used to measure nutrient intake in epidemiological studies, we acknowledge that such instruments can exhibit significant amount of measurement error that could lead to substantial bias in further analysis [38]. After exclusion of individuals who reported dietary changes, the dietary patterns remained almost identical. In the RODAM study, we performed 24hDRs for the calculation of Ghana-specific portion sizes and the average nutrient composition of some of the Ghana-FPQ food item. However, the number of 24hDRs was limited. Thus, we could not calculate age- and sex-specific portion size and nutrient compositions. Another important issue is the application of the different composition tables for the translation of food consumption into the intakes of energy and nutrients. The German Food Composition Table (BLS 3.01) (2010) and the West African Food Composition Table (2012) differ in the analytical methods and in the definition of fibre. This affects the amount of carbohydrates, because the content of dietary fibre is included in the formula, in both food composition tables. Lastly, the different nature of the exploratory methods used in the studies discussed before as well as the potential for population- and data- specific patterns make complicate the comparison of exploratory dietary patterns across different populations.

## Conclusion

In the RODAM study population, differences in dietary behaviour were consistent with the nutrition transition theory. Yet, in this West African population, traditional and indigenous foods continued to be consumed at all study sites. The identified dietary patterns were distinctly associated with age, sex, lifestyle factors, and place of residence. These results set up the basis for future studies aiming at investigating the health implications of dietary behaviour on metabolic health in the RODAM study population.

## Supplementary Material

Suppl.docx
