# Supplementary material for: Food consumption, nutrient intake, and dietary patterns in Ghanaian migrants in Europe and their compatriots in Ghana
Source: Food Nutr Res. 2017 Jul 6;61(1):1341809. doi: 10.1080/16546628.2017.1341809 (PMC5510194; doi:10.1080/16546628.2017.1341809)
Supplement: Suppl.docx [file zfnr_a_1341809_sm1815.docx]

**Suppl. Table 1**: Scores of the three dietary patterns in the three different locations. Data are shown as median (IQR)

| **Dietary pattern** | **Europe**  **(n=2011)** | **Urban Ghana (n=1429)** | **Rural Ghana (n=1103)** |
| --- | --- | --- | --- |
| “Mixed” pattern | 0.73 (-0.34; 1.21) | -0.74 (-0.94; -0.53) | -0.74 (-0.96; -0.45) |
| “Animal product” pattern | -0.19 (-0.74; 0.50) | 0.13 (-0.40; 0.79) | -0.45 (-0.92; 0.21) |
| “Traditional Ghanaian” pattern | -0.55 (-0.87; -0.11) | -0.17 (-0.49; 0.21) | 0.49 (-0.01; 1.28) |

IQR: Interquartile range

**Suppl. Table 2:** Nutrient density values of micronutrient intake across quintiles of adherence to the “mixed” dietary pattern. Data are shown as median (IQR).

|  | **Q1 (n=781)** | **Q2 (n=781)** | **Q3 (n=781)** | **Q4 (n=781)** | **Q5 (n=781)** |  |
| --- | --- | --- | --- | --- | --- | --- |
| **Micronutrients** | **Median (IQR)** | **Median (IQR)** | **Median (IQR)** | **Median (IQR)** | **Median (IQR)** | ***p* for trend** |
| Dietary fibre (g/1000 kcal) | 14.2 (4.0) | 14.8 (3.7) | 14.7 (4.7) | 13.7 (3.5) | 14.2 (4.3) | 0.002 |
| Ca (mg/1000 kcal ) | 214.2 (49.8) | 221.6 (51.3) | 239.2 (52.1) | 274.1 (66.8) | 296.6 (77.6) | < 0.001 |
| Fe (mg/1000 kcal ) | 6.9 (1.0) | 6.9 (1.1) | 7.0 (1.2) | 7.5 (1.8) | 7.5 (1.5) | < 0.001 |
| Mg (mg/1000 kcal ) | 182.5 (29.3) | 189.3 (33.9) | 196.4 (33.7) | 206.0 (31.7) | 211.4 (32.9) | < 0.001 |
| P (mg/1000 kcal ) | 524.2 (88.5) | 526.3 (89.1) | 540.4 (98.4) | 590.3 (93.5) | 602.8 (96.3) | < 0.001 |
| Na (mg/1000 kcal ) | 1135.8 (512.1) | 1146.1 (405.6) | 1120.3 (451.9) | 1158.5 (304.1) | 1086.4 (295.5) | < 0.001 |
| K ( mg/1000 kcal) | 1742.2 (296.3) | 1802.4 (315.2) | 1864. 8 (402.2) | 2030.3 (450.3) | 2072.3 (459.1) | < 0.001 |
| Zn (mg/1000 kcal ) | 4.5 (1.3) | 4.7 (1.3) | 4.7 (1.3) | 5.3 (1.4) | 5.3 (1.2) | < 0.001 |
| Cu (mg/1000 kcal ) | 0.9 (0.2) | 0.9 (0.2) | 1.0 (0.2) | 1.1 (0.2) | 1.1 (0.2) | < 0.001 |
| Retinol (μg/day) | 9433.6 (6166.5) | 8736.2 (4563.0) | 8776.8 (4799.7) | 7034.4 (6344.8) | 6251.5 (4837.0) | < 0.001 |
| β-carotene (μg/day) | 33.3 (24.0) | 36.0 (28.8) | 48.0 (40.4) | 179.1 (235.2) | 188.0 (177.9) | < 0.001 |
| Vit. A (RE/1000 kcal ) | 835.6 (503.4) | 785.5 (391.9) | 827.7 (514.3) | 1307.7 (915.1) | 1298.7 (720.2) | < 0.001 |
| Vit. B1 (thiamine) (mg/1000 kcal ) | 0.5 (0.2) | 0.5 (0.1) | 0.5 (0.2) | 0.6 (0.2) | 0.7 (0.2) | < 0.001 |
| Vit. B2 (riboflavin) (mg/1000 kcal ) | 0.5 (0.2) | 0.5 (0.2) | 0. 6 (0.2) | 0.7 (0.2) | 0.7 (0.2) | < 0.001 |
| Vit. B3 (niacin) (mg/1000 kcal ) | 6.7 (2.2) | 6.6 (1.9) | 6.7 (1. 9) | 8.8 (3.7) | 9.5 (3.6) | < 0.001 |
| Vit. B6 (mg/1000 kcal ) | 0.9 (0.2) | 1.0 (0.2) | 1.0 (0.2) | 1.1 (0.2) | 1.1 (0.3) | < 0.001 |
| Vit. B9 (folate) (μg/1000 kcal ) | 122.5 (29.5) | 130.5 (29.0) | 139.9 (38.7) | 160.3 (48.9) | 165.4 (44.5) | < 0.001 |
| Vit. B12 (μg/1000 kcal ) | 4.7 (5.4) | 5.6 (10.0) | 4.5 (9.7) | 2.4 (1.9) | 2.0 (1.3) | < 0.001 |
| Vit. C (mg/1000 kcal ) | 85.1 (66.2) | 122.2 (75.2) | 121.6 (72.5) | 97.8 (47.0) | 106.1 (50.8) | 0.471 |
| Vit. D (μg/1000 kcal ) | 2.8 (2.5) | 2.4 (1.8) | 1.9 (1.6) | 1.4 (1.3) | 1.3 (1.1) | < 0.001 |
| Vit. E (mg/1000 kcal ) | 7.9 (2.2) | 7.7 (2.2) | 7.5 (2.2) | 8.4 (2.3) | 8.3 (2.3) | < 0.001 |

IQR: Interquartile range

**Suppl. Table 3:** Nutrient density values of micronutrient intake across quintiles of adherence to the “rice, pasta, meat and fish” dietary pattern. Data are shown as median (IQR).

|  | **Q1 (n=781)** | **Q2 (n=781)** | **Q3 (n=781)** | **Q4 (n=781)** | **Q5 (n=781)** |  |
| --- | --- | --- | --- | --- | --- | --- |
| **Micronutrients** | **Median (IQR)** | **Median (IQR)** | **Median (IQR)** | **Median (IQR)** | **Median (IQR)** | ***p* for trend** |
| Dietary fibre (g/1000 kcal) | 15.8 (4.5) | 15.1 (3.9) | 14.2 (3.7) | 13.8 (3.6) | 12.7 (3.4) | < 0.001 |
| Ca (mg/1000 kcal ) | 244.8 (84.4) | 242.7 (71.7) | 245.2 (69.8) | 245.8 (65.0) | 243.5 (63.0) | 0.153 |
| Fe (mg/1000 kcal ) | 7.0 (1.6) | 7.1 (1.4) | 7.1 (1.3) | 7.1 (1.2) | 7.1 (1.2) | 0.665 |
| Mg (mg/1000 kcal ) | 201.6 (41.3) | 201.1 (35.5) | 197.6 (33.6) | 196.6 (33.8) | 187.4 (32.4) | < 0.001 |
| P (mg/1000 kcal ) | 533.1 (131.7) | 540.5 (111.7) | 554.8 (106.7) | 565.8 (88.7) | 577.2 (79.8) | < 0.001 |
| Na (mg/1000 kcal ) | 1021.03 (355.3) | 1094.2 (342.8) | 1140.2 (359.7) | 1169.1 (394.9) | 1208.4 (436.6) | < 0.001 |
| K ( mg/1000 kcal) | 1993.9 (501.4) | 1948.3 (423.9) | 1876.4 (390.9) | 1850.9 (388.2) | 1761.4 (367.4) | < 0.001 |
| Zn (mg/1000 kcal ) | 4.4 (1.3) | 4.5 (1.2) | 4.9 (1.3) | 5.1 (1.3) | 5.5 (1.2) | < 0.001 |
| Cu (mg/1000 kcal ) | 1.0 (0.2) | 1.0 (0.2) | 1.0 (0.2) | 1.0 (0.2) | 0.9 (0.2) | < 0.001 |
| Retinol (μg/day) | 8952.2 (6375.7) | 8957.4 (4887.4) | 8039.2 (4902.5) | 7860.3 (4500.3) | 7397.6 (4769.8) | < 0.001 |
| β-carotene (μg/day) | 52.3 (115.3) | 53.4 (121.2) | 59.4 (144.6) | 58.2 (117.5) | 66.9 (140.5) | < 0.001 |
| Vit. A (RE/1000 kcal ) | 1008.6 (855.0) | 995.6 (884.8) | 912.9 (758.9) | 884.7 (768.2) | 871.3 (743.0) | < 0.001 |
| Vit. B1 (thiamine) (mg/1000 kcal ) | 0.6 (0.2) | 0.6 (0.2) | 0.6 (0.2) | 0.5 (0.2) | 0.5 (0.2) | < 0.001 |
| Vit. B2 (riboflavin) (mg/1000 kcal ) | 0.6 (0.3) | 0.6 (0.3) | 0.6 (0.2) | 0.6 (0.2) | 0.6 (0.2) | < 0.001 |
| Vit. B3 (niacin) (mg/1000 kcal ) | 6.6 (3.2) | 6.9 (2.8) | 7.4 (3.0) | 7.5 (2.8) | 8.1 (2.5) | < 0.001 |
| Vit. B6 (mg/1000 kcal ) | 1.0 (0.3) | 1.0 (0.2) | 1.0 (0.2) | 1.0 (0.2) | 1.0 (0.2) | < 0.001 |
| Vit. B9 (folate) (μg/1000 kcal ) | 150.1 (54.6) | 146.0 (50.5) | 139.4 (44.7) | 137.9 (40.1) | 131.3 (39.2) | < 0.001 |
| Vit. B12 (μg/1000 kcal ) | 2.1 (2.7) | 2.7 (3.3) | 3.3 (3.9) | 3.9 (5.4) | 4.4 (6.5) | < 0.001 |
| Vit. C (mg/1000 kcal ) | 115.0 (79.0) | 114.2 (67.4) | 106.0 (69.7) | 106.0 (60.6) | 94.7 (56.9) | < 0.001 |
| Vit. D (μg/1000 kcal ) | 1.3 (1.6) | 1.6 (1.7) | 2.0 (1.8) | 2.1 (1.8) | 2.2 (1.6) | < 0.001 |
| Vit. E (mg/1000 kcal ) | 8.0 (3.0) | 8.2 (2.6) | 8.0 (2.1) | 7.9 (2.0) | 7.7 (2.0) | 0.001 |

IQR: Interquartile range

**Suppl. Table 4:** Nutrient density values of micronutrient intake across quintiles of adherence to the “roots, tubers and plantain” dietary pattern. Data are shown as median (IQR).

|  | **Q1 (n=781)** | **Q2 (n=781)** | **Q3 (n=781)** | **Q4 (n=781)** | **Q5 (n=781)** |  |
| --- | --- | --- | --- | --- | --- | --- |
| **Micronutrients** | **Median (IQR)** | **Median (IQR)** | **Median (IQR)** | **Median (IQR)** | **Median (IQR)** | ***p* for trend** |
| Dietary fibre (g/1000 kcal) | 13.0 (3.4) | 14.0 (3.8) | 14.4 (3.9) | 14.7 (3.9) | 15.5 (4.2) | < 0.001 |
| Ca (mg/1000 kcal ) | 265.3 (68.4) | 251.9 (74.9) | 245.8 (67.8) | 237.0 (58.5) | 222.2 (59.4) | < 0.001 |
| Fe (mg/1000 kcal ) | 7.6 (1.8) | 7.1 (1.4) | 7.1 (1.2) | 7.0 (1.2) | 6.9 (1.2) | < 0.001 |
| Mg (mg/1000 kcal ) | 199.7 (39.1) | 192.3 (40.4) | 194.7 (33.7) | 197.5 (32.8) | 199.7 (31.2) | 0.318 |
| P (mg/1000 kcal ) | 583.8 (96.6) | 566.4 (106.0) | 559.8 (105.2) | 546.8 (97.3) | 522.4 (107.6) | < 0.001 |
| Na (mg/1000 kcal ) | 1198.5 (297.6) | 1186.5 (322.2) | 1165.7 (419.2) | 1076.9 (381.9) | 957.2 (328.4) | < 0.001 |
| K ( mg/1000 kcal) | 1967.7 (297.6) | 1901.5 (472.3) | 1879 (399.4) | 1838.0 (1385.6) | 1840.8 (385.0) | < 0.001 |
| Zn (mg/1000 kcal ) | 5.4 (1.6) | 5.1 (1.5) | 4.9 (1.3) | 4.7 (1.3) | 4.6 (1.2) | < 0.001 |
| Cu (mg/1000 kcal ) | 1.1 (0.2) | 1.0 (0.2) | 1.0 (0.2) | 1.0 (0.2) | 0.9 (0.2) | < 0.001 |
| Retinol (μg/day) | 7965.0 (6654.6) | 7860.7 (5525.3) | 8328.6 (5135.0) | 8605.8 (4848.3) | 8496.0 (4354.7) | < 0.001 |
| β-carotene (μg/day) | 157.4 (248.6) | 80.3 (189.6) | 54.1 (84.4) | 47.1 (46.0) | 38.1 (40.1) | 0.210 |
| Vit. A (RE/1000 kcal ) | 1385.1 (1006.9) | 1065.2 (930.7) | 899.3 (750.8) | 867.7 (575.8) | 830.4 (392.7) | < 0.001 |
| Vit. B1 (thiamine) (mg/1000 kcal ) | 0.6 (0.2) | 0.5 (0.2) | 0.5 (0.2) | 0.5 (0.2) | 0.6 (0.2) | < 0.001 |
| Vit. B2 (riboflavin) (mg/1000 kcal ) | 0.7 (0.2) | 0.7 (0.2) | 0.6 (0.2) | 0.6 (0.2) | 0.5 (0.2) | < 0.001 |
| Vit. B3 (niacin) (mg/1000 kcal ) | 8.9 (3.9) | 8.0 (3.2) | 7.4 (2.6) | 6.9 (2.2) | 6.4 (2.2) | < 0.001 |
| Vit. B6 (mg/1000 kcal ) | 1.0 (0.2) | 1.0 (0.2) | 1.0 (0.2) | 1.0 (0.2) | 1.0 (0.2) | < 0.001 |
| Vit. B9 (folate) (μg/1000 kcal ) | 153.8 (53.9) | 139.3 (49.5) | 138.3 (43.5) | 137.1 (36.4) | 139.2 (42.4) | < 0.001 |
| Vit. B12 (μg/1000 kcal ) | 2.5 (2.1) | 3.2 (3.4) | 3.8 (6.2) | 4.4 (9.2) | 3.3 (5.2) | < 0.001 |
| Vit. C (mg/1000 kcal ) | 95.6 (48.5) | 104.9 (62.5) | 108.7 (74.6) | 114.1 (69.1) | 115.4 (68.8) | < 0.001 |
| Vit. D (μg/1000 kcal ) | 1.5 (1.4) | 1.9 (1.7) | 1.9 (1.8) | 2.1 (2.0) | 1.7 (1.7) | 0.314 |
| Vit. E (mg/1000 kcal ) | 8.2 (2.3) | 8.1 (2.3) | 8.1 (2.1) | 8.0 (2.3) | 7.4 (2.3) | < 0.001 |

IQR: Interquartile range


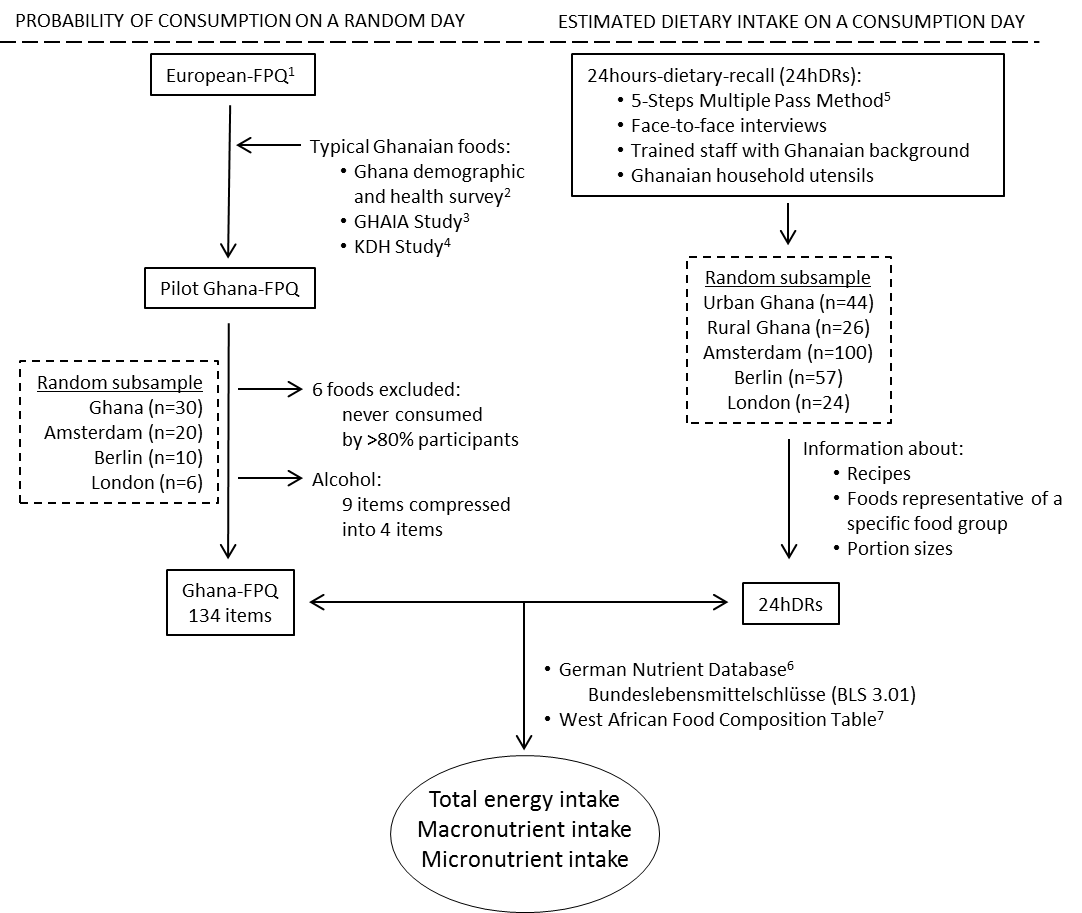


**Supplementary Figure 1**: Nutritional assessment in the RODAM Study

A semi-quantitative Ghana Food Propensity Questionnaire (Ghana-FPQ) was developed. This covers 134 items and is based on the multi-language, semi-quantitative European-Food Propensity Questionnaire (European-FPQ) (1). Typical Ghanaian food items were identified from the Ghana Demographic and Health Survey (2008) (2) and from previous studies among Ghanaians in Amsterdam (3) and in urban Ghana (4), and were included in the Ghana-FPQ. In April 2013, the Ghana-FPQ was pre-tested in 66 volunteers to assess its feasibility and acceptance within the RODAM study population. The mean duration to complete the questionnaire was 39.3 minutes (standard deviation (SD), 14.2 minutes) and the length of the Ghana-FPQ was a major complaint. In addition, the consumption of alcoholic beverages was rare and not diverse. Thus, we reduced the number of corresponding categories from nine to four. Also, we excluded six food items that were never consumed by >80% of the total pilot study population: whey cheese, sauerkraut, asparagus, swede, blood sausage and abelemamu (a fermented and boiled maize product). Finally, 134 items were covered by the Ghana-FPQ.

We also conducted 24HDRs in a random sub-sample of 251 RODAM study participants. The 24HDRs were carried out by trained staff in face-to-face interviews, according to the 5-Steps Multiple Pass Method (5). A suitcase with common Ghanaian household utensils was provided to the interviwers to facilitate the standardised quantification with familiar and uniform cooking equipment. In addition, the 24HDR provided essential information for the calculation of the average nutrient composition of Ghana-FPQ items, such as recipes and foods that are representative for a specific food group. The 24HDRs also provided information about portion sizes that were allocated to the Ghanaian foods covered in the Ghana-FPQ, separately for participants in rural Ghana, in urban Ghana, and in Europe. For the calculation of total energy intake and macro-nutrients, the Ghana-FPQ was linked to the latest versions of the German Nutrient Database (Bundeslebensmittelschlüsse (BLS 3.01)) (6) and the West-African (7) food composition tables.

1. Kaaks R, Riboli E. Validation and calibration of dietary intake measurements in the EPIC project: methodological considerations. European Prospective Investigation into Cancer and Nutrition. International journal of epidemiology. 1997;26 Suppl 1:S15-25.

2. Ghana Statistical Service, Ghana Health Service, Accra, Ghana, 2008.

3. Agyemang C, Nicolaou M, Boateng L, Dijkshoorn H, van de Born BJ, Stronks K. Prevalence, awareness, treatment, and control of hypertension among Ghanaian population in Amsterdam, The Netherlands: the GHAIA study. Eur J Prev Cardiol. 2013;20(6):938-46.

4. Frank LK, Kroger J, Schulze MB, Bedu-Addo G, Mockenhaupt FP, Danquah I. Dietary patterns in urban Ghana and risk of type 2 diabetes. Br J Nutr. 2014;112(1):89-98.

5. Conway JM, Ingwersen LA, Vinyard BT, Moshfegh AJ. Effectiveness of the US Department of Agriculture 5-step multiple-pass method in assessing food intake in obese and nonobese women. Am J Clin Nutr. 2003;77(5):1171-8.

6. Hartmann BM, Vásquez-Caicedo AL, Bell S, Krems C, Brombach C. The German nutrient database: Basis for analysis of the nutritional status of the German population. J Food Compost Anal. 2008;21:S115-S8.

7. Stadlmayr B, Charrondiere UR, Burlingame B. Development of a regional food composition table for West Africa. Food Chem. 2013;140(3):443-6.
